# Two-year longitudinal neuropsychological monitoring after unilateral and staged bilateral subthalamic nucleus deep brain stimulation

**DOI:** 10.3389/fnins.2026.1767180

**Published:** 2026-05-08

**Authors:** Szczepan Iwański, Katarzyna Polanowska, Marcin Leśniak, Michał Sobstyl

**Affiliations:** 1 2nd Department of Neurology, Institute of Psychiatry and Neurology, Warsaw, Poland; 2 Faculty of Psychology, University of Warsaw, Warsaw, Poland; 3 Department of Neurosurgery, Institute of Psychiatry and Neurology, Warsaw, Poland

**Keywords:** cognitive functions, computer-based assessment, mood, neuropsychological assessment, Parkinson’s disease, quality of life, staged deep brain stimulation, subthalamic nucleus

## Abstract

**Introduction:**

Deep brain stimulation (DBS) is an increasingly popular therapeutic method for treating motor symptoms in Parkinson’s disease, but its impact on non-motor symptoms in long-term follow-up remains debated.

**Method:**

The primary objective of this study was to monitor the cognitive functioning, mood, and quality of life in 2 years of unilateral and staged bilateral subthalamic nucleus DBS. A cohort of 30 patients was evaluated at three intervals: before DBS surgery, at 6 months, and 24 months post-surgery. The time points of neuropsychological assessments were set to control the impact of unilateral and bilateral DBS throughout the treatment. Two selected groups, unilateral and bilateral DBS, were also analyzed. The study employed a combination of computerized and paper-based tests to assess cognitive functions, alongside questionnaires to gauge emotional state and quality of life. The cognitive evaluation focused on three domains critical for daily activities: attention and processing speed, learning and episodic memory, and executive functions, including working memory and cognitive flexibility.

**Results:**

Analysis of the entire cohort from baseline through the two follow-up assessments revealed no decline in cognitive function, mood, or quality of life, alongside significant motor improvement. Additional analyses of the two subgroups—unilateral DBS and staged bilateral DBS—also showed no overall decline in any assessed domain over the 2-year follow-up period. However, comparison of cognitive outcomes with normative data indicated a higher proportion of patients meeting criteria for cognitive decline at the 24-month follow-up in the staged bilateral DBS group compared with the unilateral DBS group.

**Conclusion:**

The findings support the long-term overall stability of cognitive function, mood, and quality of life following unilateral and staged bilateral subthalamic DBS. Subgroup analyses did not reveal any significant decline in cognitive measures over time. Nevertheless, individual comparisons with normative data showed a higher proportion of patients with memory deficits in the staged bilateral DBS group after the two-year follow-up.

## 1 Introduction

Parkinson’s disease (PD) is the second most common progressive neurodegenerative disorder after Alzheimer’s disease. It is characterized not only by motor dysfunctions but also by non-motor symptoms, such as cognitive deficits and mood disturbances, which significantly affect patients’ quality of life (Stoker and Greenland, 2018). These symptoms can evolve as the disease progresses and may also be influenced by the treatments used for managing motor symptoms, such as deep brain stimulation (DBS).

DBS is an established adjunctive treatment for motor symptoms in PD, with over three decades of clinical use (Benabid et al., 1987). The most common stereotactic target for electrode implantation is the subthalamic nucleus (STN). The STN DBS is effective in managing the core symptoms of PD—tremor, bradykinesia, and rigidity—and allows for a reduction in the daily doses of dopaminergic medications (Hariz and Blomstedt, 2022), thereby mitigating their side effects. However, the STN, functionally divided into motor, associative, and limbic regions and forming part of the subcortical–frontal networks, is involved not only in motor control but also regulates cognitive and emotional functions, which can be affected by stimulation (Lo Buono et al., 2021).

Three neurosurgical approaches to DBS are commonly used: unilateral (uDBS), staged bilateral (bDBS), and simultaneous bilateral implantation. In the uDBS approach, a single lead is implanted in one hemisphere, typically in patients with predominantly unilateral symptoms. In the bDBS approach, a second lead is implanted in the contralateral hemisphere at a later time following the initial implantation, usually when symptoms on the opposite side worsen. In the simultaneous bilateral approach, all components of the DBS system are implanted during a single surgical procedure.

The most common procedure is simultaneous bilateral DBS (Alberts et al., 2008), and individual studies, review articles, and meta-analyses of neuropsychological outcomes mainly refer to this group of patients (Bucur and Papagno, 2023; Combs et al., 2015; Wang et al., 2021; Xie et al., 2016). Far fewer studies have examined uDBS and bDBS procedures, particularly those focused on behavioral consequences and long-term follow-up (Alberts et al., 2008; Cernera et al., 2020; Mikos et al., 2010; Rothlind et al., 2007; Slowinski et al., 2007; Vachez et al., 2025; Zahodne et al., 2009).

In contrast to simultaneous bilateral DBS, the uDBS and bDBS approaches are often chosen to minimize surgical risks, tailor therapy to patient response, or allow gradual symptom management. However, current evidence indicates no significant differences among these approaches in terms of postoperative complication rates, such as infection, pneumonia, hemorrhage, or overall treatment costs (Petraglia et al., 2016).

The body of literature for simultaneous bilateral DBS has been steadily growing, particularly in the past decade (Cernera et al., 2019; Lo Buono et al., 2021; Maheshwary et al., 2020; Mehanna et al., 2017; Rački et al., 2022). Despite this, formulating clear conclusions remains challenging due to variations in study methodologies, the range of functional domains assessed, differences in measurement tools, and inconsistent findings. It is even more difficult in a group of patients treated with uDBS and bDBS.

The latest meta-analysis on the long-term neuropsychological outcomes of DBS (Bucur and Papagno, 2023), which generally excluded patients with preoperative cognitive decline, suggests a mixed picture. Positive effects include mild improvements in anxiety and depression, while negative effects are observed in long-term memory and specific executive functions.

In individual studies, verbal fluency decline is the most commonly reported cognitive impairment after STN DBS (Castelli et al., 2006; Rothlind et al., 2007; Tang et al., 2015; Williams et al., 2011; Zangaglia et al., 2009), although some evidence suggests this decline may be temporary (Zangaglia et al., 2009). Findings related to attention, memory, and executive functions, however, are ambiguous. Attention and information processing speed may decrease (Mikos et al., 2010; Rothlind et al., 2007; Williams et al., 2011), although this deterioration may be transient (Tramontana et al., 2015). Memory outcomes vary, with studies reporting declines (Heo et al., 2008; Klempírová et al., 2007; Mikos et al., 2010; Tröster et al., 2017; Williams et al., 2011), stability (Castelli et al., 2010), or even improvements (Janssen et al., 2014; Tang et al., 2015). Similarly, executive function outcomes range from slightly impaired (Klempírová et al., 2007; Mikos et al., 2010; Rothlind et al., 2007; Tröster et al., 2017; Williams et al., 2011; You et al., 2020) to transiently disrupted (Tramontana et al., 2015; Yamanaka et al., 2012; Zangaglia et al., 2009), or no changes at all (Castelli et al., 2010).

Some discrepancies are found in research on mood and quality of life. Regarding mood, most studies report improvement after DBS (Castelli et al., 2006; Funkiewiez et al., 2004; Pusswald et al., 2019; Rothlind et al., 2007; Tang et al., 2015), while others find no change (Janssen et al., 2014). However, a few studies present opposite results, indicating increased symptoms of depression (Follett et al., 2010) or apathy (Funkiewiez et al., 2004). In a very recent study, Vachez et al. (2025) compared uDBS and bDBS in STN, reporting that only bilateral stimulation was associated with an increased risk of developing apathy.

Quality of life (QoL) generally improves after DBS (Jost et al., 2021; Liu et al., 2019; Smeding et al., 2011; Woods et al., 2001), although in some cases it remains unchanged (Tröster et al., 2017). Studies of unilateral STN DBS, such as those by Zahodne et al. (2009) and Slowinski et al. (2007), confirmed QoL improvement at short-term follow-up (6 months). Long-term outcomes after staged DBS were investigated by Cernera et al. (2020), who reported improvement following the first lead implantation, whereas the second lead implantation was associated with stabilization and gradual worsening over a 2-year observation period.

### 1.1 Clinical rationale for study

Given the well-documented role of the STN in both motor and non-motor functions and its status as the primary target for DBS in PD, understanding the broader impact of this intervention is crucial. Although STN DBS is an established treatment for motor symptoms, its effects on cognition, mood, and QoL remain inconsistent, with studies reporting both benefits and adverse outcomes. Particularly little is known about STN uDBS and bDBS procedures, which may be selected to tailor therapy to individual patient profiles or reduce surgical risks. Addressing this gap is essential for a comprehensive evaluation of the outcomes associated with different implantation strategies.

In this context, the present study prospectively evaluated cognitive and emotional functioning, as well as QoL over a 2-year period in patients with PD undergoing STN DBS. A secondary objective was to examine longitudinal outcomes in two selected groups: uDBS and bDBS. Additionally, we compared key cognitive outcomes with normative data to determine the proportion of patients meeting criteria for cognitive decline at each assessment time point in both groups.

## 2 Materials and methods

### 2.1 Participants

This study was conducted in accordance with the Declaration of Helsinki and was approved by the Ethical Committee of the Institute of Psychiatry and Neurology, Warsaw, Poland (no. 30/2018). All patients provided written informed consent prior to enrollment in the study.

Participants were patients with idiopathic PD, admitted to the Department of Neurosurgery, Institute of Psychiatry and Neurology, between July 2018 and March 2022, who met the following inclusion criteria: (1) age between 50 and 70 years, (2) at least 5 years since the PD diagnosis (criteria of the Parkinson’s Disease Society; Postuma et al., 2015), (3) a stable and optimal regimen of dopaminergic medication for at least 1 month before study entry, and (4) a favorable response to levodopa (>30% improvement) on the Unified Parkinson’s Disease Rating Scale Part III: motor assessment (UPDRS-III). Exclusion criteria were as follows: (1) diagnosis of secondary or atypical parkinsonism, (2) history of any other clinically significant neurological disorder (e.g., head trauma, stroke, brain tumor, or epilepsy) or psychiatric disorders (major depressive or bipolar disorder, psychotic disorders, and alcohol or substance use disorder), (3) neurobehavioral contraindication to DBS or conditions limiting its therapeutic efficacy, as identified during neuropsychological assessment (e.g., dementia and significant dysexecutive syndrome), (4) general contraindications to neurosurgery, and (5) severe movement disorders interfering with the performance of cognitive tests.

**Figure 1:**
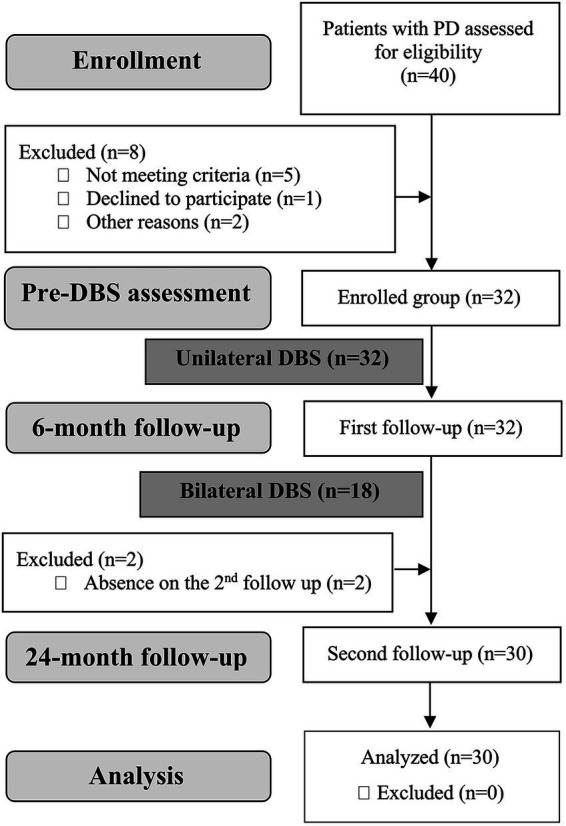
Patient flow diagram.

The sample size was calculated based on the effect size reported in the meta-analysis by Perestelo-Pérez et al. (2014) using G*Power software (Faul et al., 2007). To achieve 80% statistical power in a repeated-measures mixed design, a minimum of 26 participants was required. However, the reported effect size (Hedges’ g = 0.53) pertains only to UPDRS-III and not to non-motor outcome measures, for which effect sizes range from 0.04 to 0.56. Ultimately, 33 patients were enrolled in the study, of which 30 completed all planned procedures. A diagram of patient flow throughout the study is shown in Figure 1.

The group of 30 patients was further divided into two groups: uDBS (*n* = 14) and bDBS (*n* = 16). At the 6-month follow-up, all patients had undergone uDBS; therefore, this time point was used to evaluate short-term postoperative changes. The 24-month follow-up allowed comparisons across baseline, 6-month, and 24-month follow-up assessments.

### 2.2 Study procedure

The study consisted of the following phases: (1) screening for eligibility, (2) pre-DBS assessment just before surgery (baseline assessment), (3) initial unilateral STN DBS related to dominant movement symptoms, (4) 6-month follow-up, (5) second-stage STN DBS in patients with clinical indications, resulting in bilateral stimulation, and (6) 24-month follow-up. Follow-up assessments were scheduled to allow a minimum 6-month interval after each STN DBS procedure (uDBS and bDBS) for recovery from potential microlesion effects related to intracranial electrode implantation, while keeping the interval between visits no longer than 24 months to minimize the influence of aging and disease progression, consistent with follow-up intervals commonly reported in long-term neuropsychological studies (e.g., Bucur and Papagno, 2023).

#### 2.2.1 Screening for eligibility

To assess the dominant form and severity of PD motor symptoms in a drug-free state (at least 12 h without antiparkinsonian medications—MedOFF) and response to levodopa (MedON), patients were assessed twice using the UPDRS-III, which includes subscales for bradykinesia, tremor, rigidity, and axial symptoms.

To exclude serious cognitive and depressive disorders, patients were assessed using the Polish versions of the Addenbrooke’s Cognitive Examination – Third Edition (ACE-III; Senderecka et al., 2014) and the Beck Depression Inventory – Second Edition (BDI-II; Beck et al., 1996, Polish version, Łojek and Stańczak, 2019) using cut-off points of 82 (out of 100) and 14 (out of 63), respectively. Since some items in the BDI-II scale show somatic correlation, if the cut-off point for depressive symptoms was slightly exceeded, an in-depth clinical interview was conducted to confirm or exclude depression.

For patients enrolled in the study, UPDRS-III, ACE-III, and BDI-II scores, collected in the MedON state, were used to characterize the PD group at baseline. The demographic and clinical characteristics of study participants are shown in Table 1.

**Table 1:** Demographic and clinical characteristics of patients with Parkinson’s disease.

Study participants	Scores
Number of patients, n	30
Sex (male/female), n	20/10
Age, years, mean (SD)	62.84 (7.03)
Education, years, mean (SD)	13.39 (3.68)
Length of PD, years, mean (SD)	8.97 (3.07)
PD dominant form (akinetic-rigid/tremor/mixed), n	11/10/12
Motor symptoms (UPDRS-III), mean (SD)	27.71 (8.29)
Levodopa equivalent daily dose, mg/day (range in mg/day)	840 (250–1,600)
General cognition, (ACE-III), mean (SD)	90.58 (7.52)
Mood and Emotional state (BDI-II), mean (SD)	9.71 (6.82)

n, values in numbers; SD, standard deviation; UPDRS-III, the Unified Parkinson’s Disease Rating Scale-Part III; ACE-III, Addenbrooke’s Cognitive Examination-Third Edition; BDI-II, the Beck Depression Inventory-Second Edition.

#### 2.2.2 Surgical procedure

Depending on the laterality of dominant motor symptoms, patients underwent uDBS, and, in a subset of patients when clinically indicated (e.g., due to insufficient control of contralateral motor symptoms or disease progression), contralateral bDBS lead implantation was subsequently performed. Each unilateral subthalamic electrode implantation was performed under local anesthesia in the MedOFF state. The Leksell G stereotactic head frame was secured to the patient’s skull, and stereotactic contrast-enhanced CT images were obtained. These images were then merged with preoperative MRI using stereotaxic surgical trajectory planning software.

A permanent quadripolar DBS electrode (Model 3,389, Medtronic, Minneapolis, United States) was implanted along the preplanned stereotactic trajectory. The stereotactic target constituted the postero-lateral (motor) part of the STN in all patients. Target selection was based on T2-weighted MRI to visualize the STN and T1-weighted imaging to define a safe stereotactic trajectory with particular attention to the identification of cerebral vessels. No intraoperative microrecording was performed. Immediately after DBS lead implantation, patients underwent intraprocedural non-contrast-enhanced stereotactic CT imaging to verify the exact position of the DBS lead and to exclude intracerebral hemorrhage. If no hemorrhage was detected, the implantable pulse generator (IPG; Activa 37,603, Medtronic, Minneapolis, MN, USA) was placed during the same operative session with the patient under general anesthesia.

The generator was activated on the third postoperative day (StimON) after selecting the contact with the best therapeutic effect on motor Parkinsonian symptoms. For chronic stimulation of the STN, the initial stimulation settings were as follows: frequency 130 Hz, pulse width 60 μs, and initial voltage around 1.8 V or 2.4 V. The stimulation was monopolar, with the electrode set as the cathode and the IPG set as the anode. Patients were usually discharged home on the fifth postoperative day.

#### 2.2.3 Assessments

Neuropsychological assessments were performed during the best clinical state, that is, in the MedON state before DBS procedure and in the MedON/StimON state after DBS implantation and initiation of stimulation.

All patients were assessed for crucial cognitive functions, mood, QoL, and relevant clinical characteristics (DBS-related motor symptoms and adverse events, levodopa equivalent daily dose) using the same neuropsychological and neurological methods, applied in the same order.

Cognitive functions were assessed using six computer-based tasks from the Cambridge Neuropsychological Test Automated Battery (CANTAB; Sharma, 2013). These methods were selected for their ability to provide automated administration and real-time scoring, minimize examiner-related variability and errors, and particularly offer high sensitivity in detecting subtle changes in cognitive performance while using alternate versions (Henkel et al., 2025; Zygouris and Tsolaki, 2015). The administered tasks, targeting attention, processing speed, non-verbal memory, and executive function, included the Motor Screening Task (MOT), Reaction Time (RTI), Rapid Visual Processing (RVP), Paired Associates Learning (PAL), Pattern Recognition Memory (PRM), and Multitasking Test (MTT). All computerized tasks were preceded by a brief training phase, and alternate parallel forms of memory tests were used to minimize practice effects. The potential influence of DBS-related motor changes on cognitive performance was controlled using psychomotor speed measures from MOT.

In addition to the computerized measures, two paper-based tests were administered: alternate versions of the Rey Auditory Verbal Learning Test (RAVLT; Bean, 2011), including two indicators – learning, defined as the sum of correctly recalled words across the first five consecutive trials (RAVLT-L) and delayed recall (RAVLT-D) – and the Digit Span subtest from the Wechsler Adult Intelligence Scale (Brzeziński et al., 2011). Since the full cognitive assessment lasted approximately 90 min, it was divided into two sessions with a 15-min break to reduce fatigue.

To assess mood and QoL, the BDI-II and the Parkinson’s Disease Questionnaire (PDQ-39; Jenkinson et al., 1997) were used, respectively. A description of all neuropsychological tests used is presented in Supplementary Table 1.

To compare changes in motor functioning before and after DBS surgery, the UPDRS-III was repeated at subsequent time points. To minimize DBS-related variables, initial stimulation parameters, except voltage, were constant and unchanged throughout the entire observation. The therapeutic effect of DBS was evaluated at each follow-up visit.

### 2.3 Statistical analyses

Data analysis was performed using R (v. 4.5.2) within the RStudio environment (Posit Team, 2026). The distribution of continuous variables was examined using the Shapiro–Wilk test, skewness and kurtosis indices, and visual inspection of density plots. Baseline comparisons between the uDBS and bDBS groups were conducted using independent-samples *t*-tests, whereas categorical variables were analyzed using χ^2^ tests. All baseline comparisons were two-sided, and effect sizes were reported as Cohen’s d.

Although scores of several measures at individual time points deviated slightly from normality, skewness and kurtosis values remained within acceptable ranges (absolute values < 2.0). Therefore, linear mixed-effects models (LMMs) were used for the primary analyses assessing post-DBS motor, cognitive, and behavioral outcomes. These models were implemented using the *lme4* (v. 1.1–38) and *lmerTest* (v. 3.2–0) packages in R.

The selection of the appropriate model for each outcome variable followed a multistep procedure. First, data were examined for linearity and potential interactions by comparing a linear model with a quadratic model to detect possible curvature in the trajectories over time. Both models included fixed effects of group (uDBS vs. bDBS), time (pre-DBS, 6-month follow-up, and 24-month follow-up), and their interaction, with a random intercept for participants to account for within-subject dependence. Models were compared using likelihood-ratio tests (ANOVA), and χ^2^ statistics, AIC, and BIC values were used to determine which model provided the best fit.

Based on the presence or absence of interaction effects and the form of the temporal trend, one of the following model types was selected (see also Supplementary Table 2):

1. A linear model including all participants as a single group (uDBS + bDBS), if a linear trend was confirmed and no group × time interaction was present.
2. A linear model including two groups (uDBS vs. bDBS), if a linear trend and a significant group × time interaction were confirmed.
3. A model with custom contrasts for time, including all participants (uDBS + bDBS), if a quadratic trend was confirmed and no group × time interaction was present.
4. A model with custom contrasts for time, including two groups (uDBS vs. bDBS), if both a quadratic trend and a significant group × time interaction were confirmed.

For contrast analyses, estimated marginal means were calculated for each time point.

Finally, covariates were added to the selected model when appropriate. Age, years of education, and duration of Parkinson’s disease were considered potential covariates due to their known associations with motor, cognitive, and behavioral outcomes, and the presence of numerical differences between groups at baseline. Pre-DBS MOT performance was additionally considered as a covariate for cognitive tests involving manual responses (PRM, MTT, RTI, PAL, and RVP). Covariates were evaluated by adding them sequentially to the LMMs, and model fit was compared using likelihood-based analyses of deviance. For each outcome measure, the most parsimonious model providing an adequate fit to the data was selected for inference. If the inclusion of a second covariate worsened model fit, the simpler model including only one covariate was retained based on comparisons of AIC values.

## 3 Results

Between-group comparisons at baseline (i.e., pre-DBS) revealed only differences in one demographic variable (years of education, Table 2) and one clinical variable (depressive symptoms). Specifically, the uDBS group exhibited lower educational attainment and greater depressive symptom severity.

**Table 2:** Demographic and baseline clinical characteristics of subgroups uDBS and bDBS.

Variable	uDBS (*n* = 14)	bDBS (*n* = 16)	chi^2^/t	*p*	d
Sex (women), *n* (%)	5 (36%)	5 (31%)	0.017	0.9	–
Age (years), mean (SD)	61.9 (6.5)	63.6 (7.8)	−0.65	0.522	–
Education (years), mean (SD)	11.6 (2.9)	15.1 (3.7)	−2.84	0.008	1.02
Length of PD (years), mean (SD)	8.3 (2.6)	9.8 (3.4)	−1.34	0.191	–
UPDRS	43.4 (13.4)	51.8 (12.7)	−1.75	0.09	–
ACE-III, mean (SD)	90.6 (7.9)	90.5 (7.7)	0.05	0.96	–
BDI-II	13.6 (8.4)	7 (4.2)	2.43	0.03	1.07

uDBS, unilateral Deep Brain Stimulation; bDBS, staged bilateral Deep Brain Stimulation; ACE-III, Addenbrooke’s Cognitive Examination-Third Edition; BDI-II, Beck Depression Inventory- Second Edition; UPDRS, Unified Parkinson’s Disease Rating Scale.

The neuropsychological assessment scores at three time points, illustrating performance changes over 2 years of observation and displaying the proportion of uDBS versus bDBS at each time point, are presented in Supplementary Table 3.

**Figure 2:**
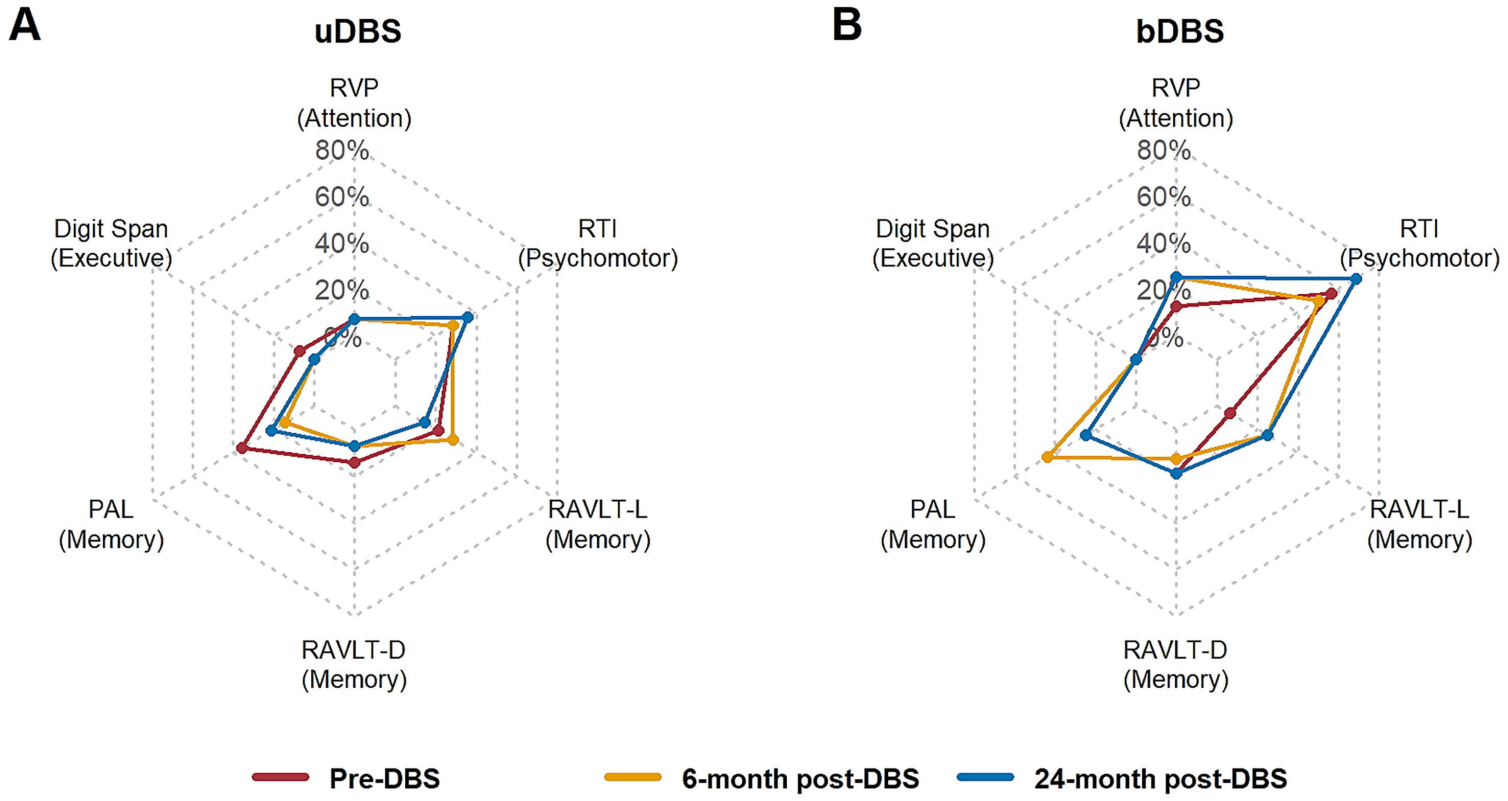
Patients performing below normative thresholds (−2 SD) in cognitive tests across assessments. **(A)** unilateral DBS (uDBS); **(B)** staged bilateral DBS (bDBS). RTI, Reaction Time; RVPA, Rapid Visual Information Processing; PAL, Paired Associates Learning; RAVLT-L, Rey’s Auditory Verbal Learning Test – Learning (the sum of correctly recalled words across the first five consecutive trials); RAVLT-D, Rey’s Auditory Verbal Learning Test – Delayed recall.

In addition, we compared the main cognitive outcomes with normative values at baseline and at both follow-up assessments (see Supplementary Table 4) to determine how many patients were below the normative range (see Figure 2) and how many were within or above the normative values during the observation period (see Supplementary Table 5).

The z-scores for the main cognitive outcomes for all participants were calculated using each participant’s score, the normative group’s mean, and standard deviation. All scores equal to or below 2 SD are interpreted as below the norm, equal to or above 2 SD as above the norm, and all other scores as within the norms.

In the bDBS group, more patients (56%) showed deficits in the psychomotor outcome (RTI) compared with the uDBS group (29%). This observation is consistent with a statistical trend of baseline differences in neurological symptoms, as measured by the UPDRS. Conversely, more patients in the uDBS group (21%) exhibited deficits in one of the verbal memory indicators (RAVLT-L) compared with the bDBS group (6%). These differences were not detected in statistical comparisons. Other outcomes in memory, attention, and executive functions were similar between groups.

### 3.1 Motor functions post-DBS

Descriptively, the largest reduction in neurological symptoms was observed at the 6-month follow-up (approximately 30% on average). At the 24-month follow-up, symptoms remained reduced by approximately 24% compared with the pre-DBS assessment. Consistent with this pattern, the LMM analysis revealed a non-linear relationship between time and UPDRS scores with two distinct slopes: a decrease in scores between pre-DBS and the 6-month follow-up, indicating symptom improvement (*β* = −15.69, 95% CI [−19.24, −12.13], *t*(56) = −8.84, *p* < 0.001), and an increase between the 6- and 24-month follow-ups (*β* = 5.33, 95% CI [1.77, 8.88], *t*(56) = 3.00, *p* = 0.004).

**Figure 3:**
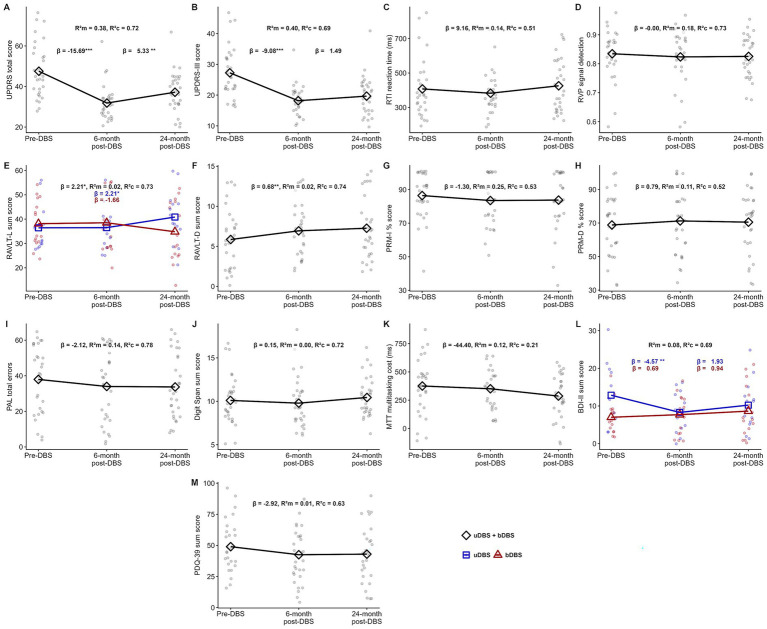
Linear mixed effects model results across pre- and post-DBS assessments. Panels **A–M** represent individual measures. Each plot displays the results of linear mixed effects models. The top annotation shows overall model statistics (*β* coefficients for the fixed effects of time are reported for linear models only). Annotations below indicate either group-specific linear trends (centered) or slope estimates for each interval (pre-DBS to 6-month post-DBS and 6-month to 24-month post-DBS). uDBS, unilateral Deep Brain Stimulation; bDBS, bilateral Deep Brain Stimulation; BDI-II, Beck Depression Inventory – Second Edition; MTT, Multitasking Test; PAL, Paired Associates Learning; PDQ-39, Parkinson’s Disease Questionnaire; PRM-I, Pattern Recognition Memory - Immediate; PRM-D, Pattern Recognition Memory – Delayed; RAVLT-L, Rey’s Auditory Verbal Learning Test – Learning (the sum of correctly recalled words across the first five consecutive trials); RAVLT-D, Rey’s Auditory Verbal Learning Test – Delayed recall; RTI, Reaction Time; RVP, Rapid Visual Information Processing; UPDRS, Unified Parkinson Disease Rating Scale; R^2^m: marginal R^2^ (variance explained by fixed effects); R^2^c: conditional R^2^ (variance explained by fixed and random effects). Significance levels: * *p* < 0.05; ** *p* < 0.01; *** *p* < 0.001.

The group × time interaction was not statistically significant, indicating broadly comparable symptom trajectories across groups. Consequently, subsequent analyses were conducted for the entire sample. Age was included as a covariate and showed a small but statistically significant association with UPDRS scores, with slightly lower symptom severity observed in older participants (*β* = −0.45, 95% CI [−0.89, −0.01], *t*(27) = −2.02, *p* = 0.047). This model explained 72% of the variance, as indicated by the conditional *R*^2^ (Figure 3A).

Similarly, UPDRS-III scores changed non-linearly across time points, and no group × time interaction was observed. However, the only significant change was detected between pre-DBS and the 6-month follow-up (*β* = −9.08, 95% CI [−11.38, −6.77], *t*(56) = −7.88, *p* < 0.001), with no further improvement or decline at the 24-month follow-up (see Figure 3B). Age again emerged as a significant negative covariate (*β* = −0.28, 95% CI [−0.54, −0.01], *t*(27) = −2.15, *p* = 0.04).

No significant time effects were identified for RTI reaction time. However, the pre-DBS MOT score emerged as a significant positive covariate (*β* = 0.20, 95% CI [0.06, 0.34], *t*(28) = 2.60, *p* = 0.011). This linear model explained 51% of the variance and did not indicate any interaction effects (see Figure 3C).

### 3.2 Cognitive functions post-DBS

#### 3.2.1 Attention and information processing speed

No significant main or interaction effects were observed in the LMM analysis of RVP scores (see Figure 3D). The MOT score was included as a significant covariate (*β* = −0.0001, 95% CI [−0.0002, −0.00004], *t*(28) = −2.83, *p* = 0.009).

#### 3.2.2 Memory

The analysis revealed significant changes in RAVLT-L performance over time, following a linear trend. The group × time interaction was statistically significant (*β* = −3.87, 95% CI [−6.72, −1.02], *t*(58) = −2.66, *p* = 0.01), although only the uDBS group showed improvement over time (*β* = 2.21, 95% CI [0.09, 4.34], *t*(58) = 2.09, *p* = 0.04; see Figure 3E).

Similarly, RAVLT-D performance in the uDBS group improved linearly across consecutive assessments (*β* = 0.68, 95% CI [0.21, 1.15], *t*(59) = 2.85, *p* = 0.006). However, no significant main effect of group and no group × time interaction were observed (see Figure 3F). None of the potential covariates was significantly related to either RAVLT-L or RAVLT-D scores.

In the analyses of PRM immediate recall, PRM delayed recall, and PAL, none of the main fixed effects reached statistical significance. No significant interaction effects were observed, and the trajectories for both groups suggested no meaningful changes across time points (see Figures 3G–I).

#### 3.2.3 Executive functions

No significant improvement or decline was observed for Digit Span, and the group × time interaction was not statistically significant (see Figure 3J).

In the analysis of MTT performance, only a statistical trend was observed (*p* = 0.07), suggesting possible improvement over time. The pre-DBS MOT score was the only significant covariate and negatively affected MTT scores (*β* = −0.23, 95% CI [−0.39, −0.07], *t*(28) = −2.73, *p* = 0.011). However, this model showed the poorest fit among all models described, explaining only 21% of the variance (see Figure 3K).

Overall, across cognitive tasks—except for RAVLT—no consistent time effects were observed, although performance in some tests was associated with age or baseline motor covariates.

### 3.3 Mood and quality of life post-DBS

LMM analysis revealed statistically significant changes in mood across assessments. This relationship was non-linear, and the trajectories differed between groups (*β* = 1.95, 95% CI [0.001, 3.89], *t*(55.6) = 1.96, *p* < 0.05). Specifically, in the uDBS group, BDI-II scores decreased between the pre-DBS evaluation and the 6-month follow-up, indicating an improvement in mood (*β* = −4.57, 95% CI [−7.47, −1.67], *t*(54.2) = −3.16, *p* = 0.003). No further statistically significant changes were observed (see Figure 3L).

Regarding quality of life, none of the main effects were statistically significant. PDQ-39 scores remained stable across the entire study period for the whole sample (see Figure 3M).

## 4 Discussion

This cohort study addressed two primary research questions. First, it examined the longitudinal clinical effects of the whole group of unilateral DBS (uDBS) and staged bilateral DBS (bDBS) targeting the STN on cognition, mood, and QoL. Second, it compared symptom trajectories between uDBS and bDBS over a 2-year follow-up period. The findings related to each question are discussed separately below, highlighting both convergences and divergences in the observed outcomes.

The results of this observation indicated the expected improvement in neurological status, regardless of the DBS approach, with the greatest motor improvement observed 6 months after implantation of the initial electrode, after controlling for age.

With respect to the cognitive and behavioral consequences of DBS, it is important to note that relatively few studies have examined the effects of unilateral and staged bilateral DBS compared with simultaneous bilateral DBS. Our findings are discussed in the context of the available data from both approaches.

Consistent with the study’s aims, observation across the entire cohort of uDBS and bDBS patients revealed no significant decline in cognitive, mood, or QoL measures. Outcomes remained stable at both the 6- and 24-month follow-up, even after adjusting for age, education, disease duration, and baseline motor speed. These findings are encouraging and align with previous reports on simultaneous bilateral STN DBS.

Neither DBS procedure had a significant impact on sustained attention, processing speed, or aspects of executive function, such as working memory and inhibition, even though these domains were assessed using computer-based tests, allowing precise measurement of reaction times.

Our findings are consistent with some meta-analyses (Bucur and Papagno, 2023; Wang et al., 2021; Elkin et al., 2021; Xie et al., 2016), although others report a slight decline (Combs et al., 2015; Parsons et al., 2006). Specifically for executive functions, more evidence points to a mild deterioration (Bucur and Papagno, 2023; Combs et al., 2015; Parsons et al., 2006; Xie et al., 2016) than to stability (Wang et al., 2021; Elkin et al., 2021).

Similarly, no significant adverse changes were observed in auditory–verbal learning or visual memory, which were assessed using standardized neuropsychological tests; interestingly, a slight improvement in verbal memory was noted at the 24-month follow-up. This contrasts with most evidence, which reports a small decline in memory and learning (Combs et al., 2015; Parsons et al., 2006; Wang et al., 2021; Xie et al., 2016). However, some studies observed only transient memory decline (Zangaglia et al., 2009), and a few others support our findings, suggesting that STN DBS does not negatively affect delayed recall or other aspects of memory (Contarino et al., 2007; Funkiewiez et al., 2004). Improvements in memory, although rare, have also been reported in other studies. For example, Tang et al. (2015) found higher immediate verbal memory scores at 6 months, stable for 1 year after DBS, but no significant change in delayed recall or recognition. Similarly, Janssen et al. (2014) reported a transient improvement in short-term memory during the first year, followed by a decline at 5 years.

No significant decline in mood or QoL was observed in either group; instead, BDI-II scores decreased in the uDBS group during the first 6 months post-DBS, indicating an improvement in depressive symptoms. However, this change likely reflected differences in baseline BDI-II scores, as at that stage, both groups had only one lead implanted. Most studies reported improvement in mood after STN DBS (Castelli et al., 2006; Funkiewiez et al., 2004; Pusswald et al., 2019; Tang et al., 2015; Zangaglia et al., 2009), whereas others reported no change (Janssen et al., 2014). However, there are also a few studies with opposite results, indicating increased depression symptoms (Follett et al., 2010) or apathy (Funkiewiez et al., 2004). In our study, only one patient (3%) reported a significant increase (BDI-II) in symptoms of depression. Depression following DBS treatment does not appear to be a clinical concern, provided that eligibility criteria exclude individuals with depression and the treatment is administered without serious side effects. Similarly, most studies to date have reported an improvement in QoL (Jost et al., 2021; Smeding et al., 2011; Zangaglia et al., 2009). Liu et al. (2019) reported that just over half of the patients improved in this regard, approximately two-fifths showed no change, and fewer than one in ten experienced a decline following DBS. Elkin et al. (2021) studied predictors for QoL 3 years after DBS and found that it depended on baseline neuropsychological and neuropsychiatric non-motor symptoms. Exploring these predictors may help identify strategies to improve the QoL for patients after DBS. Nevertheless, baseline cognitive status was not significantly associated with QoL outcomes in this study.

Further observations of the uDBS and bDBS groups in the context of longitudinal analyses indicated generally similar outcomes, while also revealing some differences in disease progression trajectories. The two groups performed similarly at subsequent follow-up assessments, with the exception of verbal learning, for which the slope of improvement was slightly steeper in the uDBS group. These findings are not consistent with those of Mikos et al. (2010), who assessed patients after uDBS in the STN and reported a decline in processing speed, or with those of Rothlind et al. (2007), who observed a decline in working memory after the first procedure, with a further decrease following the second. In contrast, Slowinski et al. (2007) reported improved mental flexibility after such surgery.

Although the bDBS group exhibited lower scores on the BDI-II, a measure of depressive symptom severity, and a flatter mood trajectory over time, the group as a whole did not show any worsening after the second implantation. QoL did not differ between the uDBS and bDBS groups. Vachez et al. (2025) found that bDBS in STN may promote apathy compared with uDBS. More consistent results concern QoL, which most frequently improves after uDBS (Cernera et al., 2020; Slowinski et al., 2007; Woods et al., 2001; Zahodne et al., 2009). In turn, Cernera et al. (2020), using a bDBS protocol similar to ours, demonstrated improvement after the first lead implantation, stabilization after the second, and subsequent gradual worsening, concluding that the improvement in QoL was primarily driven by the first lead.

In addition to the LMM analyses, we compared individual results with normative data to determine the proportion of patients who met criteria for impairment, remained within the normal range, or scored above the normative mean at baseline and at the two follow-up assessments. Over the 2-year follow-up period, fewer patients in the uDBS group achieved above-norm scores on memory measures (visuospatial and verbal) compared with baseline and the 6-month follow-up. Additionally, for the psychomotor measure, only one more patient performed below the normative range at 24 months compared to all previous assessments.

In the bDBS group, a higher proportion of patients scored below the normative range in psychomotor speed, attention, and verbal learning, particularly when comparing pre-DBS assessment with the 24-month follow-up. From a clinical perspective, these findings may suggest a more pronounced progression of disease-related cognitive changes in the bDBS group.

These observations indicate that even in the absence of statistically significant differences at follow-up, careful monitoring of non-motor symptoms is warranted at each stage of DBS treatment. Notably, differences observed between groups at baseline suggest that patients who ultimately underwent bDBS may have differed clinically, in terms of disease progression, from those treated with uDBS.

The strategy of initiating treatment with uDBS and considering a second implantation based on the evolving clinical course reflects the heterogeneity of disease progression. Furthermore, our findings contribute to the ongoing debate as to whether simultaneous bilateral DBS is invariably superior to uDBS, suggesting that a more individualized treatment approach may be warranted.

### 4.1 Strengths and limitations

One of the strengths of our study is that the cognitive assessment was primarily computer-based, which is still not a common practice in this clinical population (Albano et al., 2022; Astalosch et al., 2024). Computer-based tests enable precise measurement of reaction time and movement accuracy, particularly in motor and attentional tasks, while reducing the impact of motor deficits on cognitive performance. In our study, we found no changes in simple reaction time or motor precision, suggesting no significant influence of motor improvement on attention and executive function outcomes. This is important, as improvements in motor function during cognitive testing could potentially mask subtle changes in cognitive performance. Our results indicate that motor improvement reflected in UPDRS Part III scores may not be sufficient to control for performance in cognitive tests. Therefore, we emphasize the need to include motor measures analogous to those used in cognitive tasks.

DBS is a highly precise neurosurgical technique that targets structures within millimeters of specific anatomical landmarks and is therefore likely to have minimal effects on other brain circuits, although the STN is a key node within several functional networks. Consequently, DBS does not appear to cause broad impairment in cognitive or affective functions related to specific cortico-subcortical networks. However, it may still be associated with changes in selected domains, such as attention and processing speed, as observed in our study. From a neuropsychological perspective, different tests vary in sensitivity, and computer-based tools may capture subtle performance changes more precisely than traditional paper-and-pencil measures.

One limitation of the present study is the absence of a control group of PD patients treated pharmacologically without DBS, which would have enabled comparison of DBS-related changes with the natural progression of the disease. To partially account for disease progression, follow-up assessments were conducted over 2 years, with the first follow-up scheduled 6 months after DBS to minimize the transient effects of surgery and stimulation parameter adjustments. We also compared primary cognitive outcomes with normative data to monitor the proportion of patients who met criteria for impairment, remained within the normal range, or performed above normative expectations during the 2-year observation period.

The second important limitation is the relatively small sample size in the context of the marked biological, clinical, and cognitive heterogeneity of Parkinson’s disease, which may limit generalizability and necessitate cautious interpretation of the results.

## 5 Conclusion

Our findings generally support the safety of unilateral and staged bilateral STN DBS with respect to non-motor outcomes, such as cognition, mood, and quality of life. Nevertheless, some differences in 2-year outcomes were observed between the uDBS and bDBS groups, favoring the uDBS group, particularly in the memory domain. This observation was even more pronounced from a clinical perspective based on individual comparisons with normative data. Given the limitations of our study, further research examining the neuropsychological consequences of uDBS and staged bDBS is warranted.

## Data Availability

The raw data supporting the conclusions of this article will be made available by the authors, without undue reservation.
