# Supplementary material for: Two-year longitudinal neuropsychological monitoring after unilateral and staged bilateral subthalamic nucleus deep brain stimulation
Source: Front Neurosci. 2026 May 8;20:1767180. doi: 10.3389/fnins.2026.1767180 (PMC13194385; doi:10.3389/fnins.2026.1767180)
Supplement: Supplementary file 1 [file Table_1.DOCX]

Table 1. Description of all neuropsychological tests used

| **Assessed domains** | | | **Tests** | **Tests descriptions** |
| --- | --- | --- | --- | --- |
| **Cognition** | **psychomotor speed** | | **MOT** | **Motor Screening Task** (computer test)   - a patient is asked to quickly touch the cross displayed on a different location on the screen |
|  |  |  | **RTI** | **Reaction Time** (computer test)   - a patient is asked to quickly touch one lights up circle from five circles |
|  | **attention and information processing speed** | | **RVP** | **Rapid Visual Information Processing** (computer test)   - a sequence of rapidly changing digits is presented on the screen, among which the patient must detect three predefined sets of digits and quickly react to them |
|  | **memory** | visuo-spatial memory | **PAL** | **Paired Associates Learning** (computer test)   - sets of boxes are opened in random order to reveal places of different designs; when equivalent patterns are then displayed individually, the patient must indicate the location of each previously presented pattern |
|  |  | recognition – immediate | **PRM-I** | **Pattern Recognition Memory-Immediate** (computer test)   - a series of hard-to-name visual patterns are presented one at a time for memorization; when, after a few minutes, they are presented in pairs with an added pattern, the patient has to recognize the previously seen patterns |
|  |  | recognition –  delayed | **PRM-D** | **Pattern Recognition Memory-Delayed** (computer test)   - a series of 12 hard-to-name visual patterns are presented one at a time for memorization; after 20 minutes delay, the patient has to recognize in pairs with an added pattern the previously seen pattern |
|  |  | auditory-verbal memory | **RAVLT** | **Rey Auditory Verbal Learning Test** (paper test)   - a patient is memorized a list of words by repeating them five times (RAVLT-learning), and then - after the second, interrupting list - these words are recalled immediately (RAVLT-immediate) and after an additional 20 minutes (RAVLT-delayed) |
|  | **executive functions** | working memory | **DS** | **Digit Span** (paper test)   - a patient must repeat an ascending series of numbers in reverse order to their presentation |
|  |  | inhibition | **MTT** | **Multitasking Test** (computer test)   - a patient must react quickly and according to a set rule to the displayed arrow, indicating the direction in which the arrow appears on the screen or specifying the side on screen (left – right) |
| **Mood** | | | **BDI-II** | **Beck Depression Inventory – Second Edition** (paper test)   - a patient is asked to answer questions regarding mood and depressive symptoms |
| **Quality of life** | | | **PDQ-39** | **Parkinson's Disease Questionnaire** (paper test)   - a patient is asked to answer questions from a self-report questionnaire regarding difficulties in such dimensions as mobility, daily leaving, communication, social situations |
