# Supplementary material for: Two-year longitudinal neuropsychological monitoring after unilateral and staged bilateral subthalamic nucleus deep brain stimulation
Source: Front Neurosci. 2026 May 8;20:1767180. doi: 10.3389/fnins.2026.1767180 (PMC13194385; doi:10.3389/fnins.2026.1767180)
Supplement: Supplementary file 2 [file Table_2.DOCX]

Table 2. Formulas for linear mixed-effects models implemented in R lmer() function (lme4 v. 1.1–38)

| Model | Formula | Comment | Outcome measure |
| --- | --- | --- | --- |
| Preliminary testing models | | | |
| Linear model (tested for linearity and group × time interactions) | score ~ group * time + (1 \| Nr) | "time" as a numerical factor; Maximum likelihood (ML) as an estimator of variance components | All (to compare with quadratic model) |
| Quadratic model (tested for curvature and group × time interactions) | score ~ group * poly(time, 2) + (1 \| Nr) | "time" as a numerical factor; Maximum likelihood (ML) as an estimator of variance components | All (to compare with linear model) |
| Final models used in analysis | | | |
| Model 1: Linear model including all participants as a single group (uDBS + bDBS) | score ~ time + covariate + (1 \| subject) | "time" as a numerical factor; Restricted maximum likelihood (REML) as an estimator of variance components | RTI, RVP, RAVLT-D, PRM-I, PRM-D, PAL, Digit Span, MTT, PDQ-39 |
| Model 2: Linear model including two groups (uDBS vs. bDBS) | score ~ group * time + (1 \| subject) | "time" as a numerical factor; Restricted maximum likelihood (REML) as an estimator of variance components | RAVLT-L |
| Model 3: Model with custom contrasts for time including all participants (uDBS + bDBS) | score ~ time + group + covariate + (1 \| subject) | "time" as a categorical factor; Restricted maximum likelihood (REML) as an estimator of variance components | UPDRS, UPDRS-III, |
| Model 4: Model with custom contrasts for time including two groups (uDBS vs. bDBS) | score ~ group * time + covariate + (1 \| Nr) | "time" as a categorical factor; Restricted maximum likelihood (REML) as an estimator of variance components | BDI-II |

BDI-II, Beck Depression Inventory – Second Edition; MOT, Motor Screening Task; MTT, Multitasking Test; PAL, Paired Associates Learning; PDQ-39, Parkinson's Disease Questionnaire; PRM-I, Pattern Recognition Memory - Immediate; PRM-D, Pattern Recognition Memory – Delayed; RAVLT-L, Rey's Auditory Verbal Learning Test – Learning (the sum of correctly recalled words across the first five consecutive trials); RAVLT-D, Rey's Auditory Verbal Learning Test – Delayed recall; RTI, Reaction Time; RVP, Rapid Visual Information Processing; UPDRS, Unified Parkinson Disease Rating Scale
