# Supplementary material for: Two-year longitudinal neuropsychological monitoring after unilateral and staged bilateral subthalamic nucleus deep brain stimulation
Source: Front Neurosci. 2026 May 8;20:1767180. doi: 10.3389/fnins.2026.1767180 (PMC13194385; doi:10.3389/fnins.2026.1767180)
Supplement: Supplementary file 3 [file Table_3.DOCX]

Table 3. Neuropsychological assessment scores at three time points for patients with unilateral DBS (uDBS) and bilateral DBS (bDBS).

| **Assessed domains** | | **Tests** | **Group** | **Pre-DBS** | **6-month post-DBS** | **24-month post-DBS** |
| --- | --- | --- | --- | --- | --- | --- |
|  |  | type |  | MedON | MedON & StimON | |
|  |  |  |  | Mean (SD) | | |
| **psychomotor speed** | | RTI –  reaction time (milliseconds) | **uDBS** | 377.04 (124.18) | 365.39 (111.20) | 434.93 (148.69) |
|  |  |  | **bDBS** | 434.38 (200.02) | 397.84 (127.45) | 418.06 (163.88) |
| **attention and information processing speed** | | RVP –  signal detection  (0.00-1.00) | **uDBS** | 0.85 (0.06) | 0.85 (0.06) | 0.85  (0.06) |
|  |  |  | **bDBS** | 0.82 (0.10) | 0.80 (0.11) | 0.80  (0.08) |
| **memory** | visuo-spatial memory | PAL –  total errors | **uDBS** | 36.93 (18.71) | 32.93 (17.86) | 31.21 (18.54) |
|  |  |  | **bDBS** | 38.94 (19.45) | 35.00 (22.47) | 36.00 (18.37) |
|  | recognition – immediate | PRM-I –  % score | **uDBS** | 87.70 (10.93) | 84.92 (10.88) | 83.33 (21.38) |
|  |  |  | **bDBS** | 85.42 (15.66) | 82.29 (16.91) | 84.37 (12.50) |
|  | recognition – delayed | PRM-D –  % score | **uDBS** | 69.64 (20.86) | 75.00  (17.12) | 73.02  (14.76) |
|  |  |  | **bDBS** | 68.23 (16.45) | 67.71  (21.70) | 68.23  (21.78) |
|  | learning | RAVLT-L – sum score | **uDBS** | 36.43 (9.26) | 36.50  (8.55) | 40.86  (10.97) |
|  |  |  | **bDBS** | 38.12 (9.38) | 38.50  (12.29) | 34.81  (12.46) |
|  | delayed recall | RAVLT-D – sum score | **uDBS** | 6.14 (3.80) | 7.43  (2.59) | 8.14  (3.32) |
|  |  |  | **bDBS** | 5.56 (3.74) | 6.44  (3.85) | 6.38  (3.98) |
| **executive functions** | working memory | Digit Span – sum score | **uDBS** | 9.07  (2.02) | 9.07  (2.97) | 10.00  (1.96) |
|  |  |  | **bDBS** | 11.12 (3.22) | 10.50  (2.88) | 10.88  (2.63) |
|  | inhibition | MTT – multitasking cost (milliseconds) | **uDBS** | 370.64  (222.45) | 330.71 (174.54) | 362.96 (125.57) |
|  |  |  | **bDBS** | 378.94 (264.58) | 368.34 (204.07) | 219.16 (221.12) |
| **Mood** | | BDI-II –  sum score | **uDBS** | 13.64 (8.37) | 8.29  (5.28) | 10.21  (6.51) |
|  |  |  | **bDBS** | 7.00 (4.23) | 7.69  (5.06) | 8.62  (6.86) |
| **Quality of life** | | PDQ-39 –  sum score | **uDBS** | 49.09 (24.72) | 42.50  (24.14) | 44.86  (24.53) |
|  |  |  | **bDBS** | 50.69 (18.71) | 42.50  (21.55) | 41.31  (21.19) |
| **DBS procedure** | | uDBS n (%) | | - | 30 (100%) | 14 (47%) |
|  |  | bDBS n (%) | | - | 0 (0%) | 16 (53%) |

BDI-II, Beck Depression Inventory – Second Edition; MOT, Motor Screening Task; MTT, Multitasking Test; PAL, Paired Associates Learning; PDQ-39, Parkinson's Disease Questionnaire; PRM-I, Pattern Recognition Memory – Immediate; PRM-D, Pattern Recognition Memory – Delayed; RAVLT-L , Rey's Auditory Verbal Learning Test – Learning (the sum of correctly recalled words across the first five consecutive trials); RAVLT-D, Rey's Auditory Verbal Learning Test – Delayed recall; RTI, Reaction Time; RVP, Rapid Visual Information Process
