# Supplementary material for: Two-year longitudinal neuropsychological monitoring after unilateral and staged bilateral subthalamic nucleus deep brain stimulation
Source: Front Neurosci. 2026 May 8;20:1767180. doi: 10.3389/fnins.2026.1767180 (PMC13194385; doi:10.3389/fnins.2026.1767180)
Supplement: Supplementary file 4 [file Table_4.docx]

| Table 4. The z-scores^a^ for the main cognitive outcomes in all participants and three assessments | | | | | | | | |
| --- | --- | --- | --- | --- | --- | --- | --- | --- |
|  | | | **Psycho-motor** | **Attention** | **Memory** | | | **Executive functions** |
| **Patient**  **number** | | **Assess-ment** | RTI | RVPA | PAL | RAVLT-L | RAVLT-D | Digit Span |
| **Unilateral DBS group (uDBS)** | 1 | 1 | -1.13 | -1.41 | -2.33 ↓ | -1.65 | -2.08 ↓ | 1.33 |
|  |  | 2 | -1.75 | -1.75 | -0.64 | -2.05 | -2.08 ↓ | 1.33 |
|  |  | 3 | -1.28 | -1.64 | -1.13 | -1.65 | -1.68 | 0.66 |
|  | 2 | 1 | -1.88 | -0.99 | -0.33 | -1.37 | -0.57 | 1.00 |
|  |  | 2 | -1.23 | -1.04 | 0.71 | -1.24 | 0.1 | 1.33 |
|  |  | 3 | 0.44 | -0.52 | 0.25 | -0.58 | -0.57 | 1.00 |
|  | 3 | 1 | -1.41 | -0.88 | -2.05 ↓ | 0.04 | -0.22 | 0.00 |
|  |  | 2 | -1.04 | 0.08 | -1.28 | -0.98 | -1.03 | 0.00 |
|  |  | 3 | 0.61 | -0.28 | -2.05 ↓ | -0.16 | -0.47 | 0.00 |
|  | 4 | 1 | -1.64 | -0.15 | -0.18 | -2.08 ↓ | -0.29 | 0.00 |
|  |  | 2 | -2.33 ↓ | -0.64 | 0.05 | -0.89 | 0.00 | 0.33 |
|  |  | 3 | -2.33 ↓ | -0.28 | 0.08 | -1.25 | -0.29 | -0.33 |
|  | 5 | 1 | 0.81 | -0.81 | 0.58 | 0.04 | 0.32 | 0.66 |
|  |  | 2 | -0.05 | -0.39 | -1.75 | -0.06 | 0.05 | 0.00 |
|  |  | 3 | -0.31 | -0.74 | 0.39 | 1.06 | 1.68 | 0.66 |
|  | 6 | 1 | 0.55 | 0.92 | -0.33 | 1.13 | 1.43 | 0.66 |
|  |  | 2 | -0.92 | -0.58 | -0.47 | 0.08 | 1.43 | 2.33 ↑ |
|  |  | 3 | 1.04 | -0.50 | -0.88 | 1.79 | 1.43 | 2.00 ↑ |
|  | 7 | 1 | -2.05 ↓ | -1.48 | -0.20 | 0.89 | 0.88 | -0.33 |
|  |  | 2 | -2.33 ↓ | -0.92 | -0.50 | 0.89 | 0.00 | -1.33 |
|  |  | 3 | -2.33 ↓ | -0.77 | 0.05 | 1.25 | 1.18 | 0.00 |
|  | 8 | 1 | -0.18 | -0.99 | -0.44 | 0.55 | -0.76 | 0.33 |
|  |  | 2 | -0.08 | -0.41 | 0.44 | 0.35 | 0.32 | 0.66 |
|  |  | 3 | -0.08 | -0.71 | -0.33 | 0.65 | -0.22 | 1.33 |
|  | 9 | 1 | -2.33 ↓ | -0.77 | -2.33 ↓ | -2.16 ↓ | -2.57 ↓ | -0.33 |
|  |  | 2 | -1.13 | -0.61 | -1.88 | -2.82 ↓ | -1.57 | -0.33 |
|  |  | 3 | -0.64 | -0.71 | -1.34 | -2.29 ↓ | -1.23 | 0.33 |
|  | 10 | 1 | 1.23 | -0.05 | -1.08 | -0.98 | -0.76 | -0.33 |
|  |  | 2 | 0.77 | 0.05 | -1.48 | 0.14 | -0.22 | -1.00 |
|  |  | 3 | 1.17 | -0.28 | -0.77 | 0.86 | 0.32 | 0.00 |
|  | 11 | 1 | -1.88 | -1.28 | -1.41 | -0.31 | -1.38 | -0.66 |
|  |  | 2 | -2.33 ↓ | -1.08 | -1.88 | -0.31 | 0.54 | 0.66 |
|  |  | 3 | -2.33 ↓ | -1.23 | -0.95 | -0.55 | 0.15 | 0.66 |
|  | 12 | 1 | -2.33 ↓ | -1.08 | -1.75 | -1.61 | -0.62 | 0.00 |
|  |  | 2 | -1.55 | -1.13 | -0.05 | -2.51 ↓ | -0.14 | -0.66 |
|  |  | 3 | -1.75 | -2.05 ↓ | 0.23 | 0.67 | 1.29 | 0.66 |
|  | 13 | 1 | -2.33 ↓ | -1.48 | -2.33 ↓ | -0.98 | -1.57* | -2.00 ↓ |
|  |  | 2 | -2.33 ↓ | -1.64 | -2.33 ↓ | 0.14 | 0.05 | -1.66 |
|  |  | 3 | -2.33 ↓ | -1.88 | -2.33 ↓ | 0.04 | 0.05 | -0.33 |
|  | 14 | 1 | -1.75 | -2.05 ↓ | -2.33 ↓ | -2.72 ↓ | -1.68 | 1.33 |
|  |  | 2 | -0.88 | -2.05 ↓ | -2.33 ↓ | -2.32 ↓ | -1.28 | -0.66 |
|  |  | 3 | -2.33 ↓ | -1.13 | -2.33 ↓ | -3.12 ↓ | -2.05 ↓ | 0.66 |
| **Bilateral DBS group (bDBS)** | 15 | 1 | 0.31 | -0.52 | -1.64 | -0.05 | -1.68 | 1.33 |
|  |  | 2 | -1.08 | -0.58 | -2.33 ↓ | 0.08 | -1.28 | 0.66 |
|  |  | 3 | -0.39 | -0.74 | -1.88 | 0.21 | -1.28 | 1.33 |
|  | 16 | 1 | -1.75 | -0.50 | -0.95 | -0.58 | -0.90 | 0.00 |
|  |  | 2 | -2.33 ↓ | -1.08 | -1.28 | -2.29 ↓ | -1.23 | 0.00 |
|  |  | 3 | -2.33 ↓ | -1.75 | -0.33 | -2.29 ↓ | -0.90 | 1.00 |
|  | 17 | 1 | -2.33 ↓ | -0.55 | -1.34 | -1.52 | -2.08 ↓ | 0.66 |
|  |  | 2 | -1.88 | -1.34 | 0.05 | 0.35 | -0.48 | 0.00 |
|  |  | 3 | -2.33 ↓ | -1.48 | -0.81 | 0.48 | 1.12 | 0.00 |
|  | 18 | 1 | -0.08 | -0.50 | -1.88 | -0.67 | -1.30 | 2.66 ↑ |
|  |  | 2 | -1.34 | -0.99 | -1.88 | -0.16 | -1.03 | 0.66 |
|  |  | 3 | -0.67 | -1.08 | -1.75 | -1.29 | -1.03 | 1.33 |
|  | 19 | 1 | -2.33 ↓ | -1.41 | 0.33 | 0.47 | 0.43 | 0.66 |
|  |  | 2 | -0.36 | -0.64 | 0.99 | 1.13 | 1.77 | 0.33 |
|  |  | 3 | -0.64 | -0.44 | 0.15 | 0.08 | 1.43 | 0.00 |
|  | 20 | 1 | -2.33 ↓ | -1.75 | -2.33 ↓ | -1.45 | -3.00 ↓ | -0.33 |
|  |  | 2 | -2.33 ↓ | -2.33 ↓ | -2.33 ↓ | -2.06 ↓ | -2.52 ↓ | -0.66 |
|  |  | 3 | -2.33 ↓ | -2.33 ↓ | -2.33 ↓ | -2.36 ↓ | -2.05 ↓ | 0.33 |
|  | 21 | 1 | -2.33 ↓ | -0.41 | -0.39 | 0.75 | 1.67 | 1.66 |
|  |  | 2 | -2.33 ↓ | -0.55 | -2.05 ↓ | 1.26 | 1.67 | 1.33 |
|  |  | 3 | -2.33 ↓ | -0.81 | -0.50 | 1.85 | 3.20 ↑ | 2.33 ↑ |
|  | 22 | 1 | 0.84 | -0.95 | -2.33 ↓ | -0.67 | -1.30 | 1.33 |
|  |  | 2 | -0.67 | -1.28 | -1.48 | -0.98 | -1.30 | 1.33 |
|  |  | 3 | 0.88 | -1.41 | -1.48 | -1.04 | -1.00 | 1.33 |
|  | 23 | 1 | -2.33 ↓ | -0.84 | -0.74 | 0.55 | 0.32 | 1.00 |
|  |  | 2 | -2.33 ↓ | -0.67 | -2.33 ↓ | 0.24 | -0.22 | 0.00 |
|  |  | 3 | -2.33 ↓ | -1.55 | -0.50 | 0.35 | 0.32 | 0.66 |
|  | 24 | 1 | -1.88 | 0.15 | -0.61 | 1.16 | 0.05 | 2.33 ↑ |
|  |  | 2 | -1.75 | 0.67 | 0.52 | 1.77 | 1.40 | 3.00 ↑ |
|  |  | 3 | -1.75 | 0.36 | -0.39 | 0.75 | 0.59 | 2.33 ↑ |
|  | 25 | 1 | -2.33 ↓ | -0.47 | -0.95 | -1.64 | -1.03 | 0.00 |
|  |  | 2 | -2.33 ↓ | -1.34 | 0.13 | -2.20 ↓ | -1.64 | -0.66 |
|  |  | 3 | -2.33 ↓ | -1.28 | 0.03 | -0.04 | -1.33 | 0.33 |
|  | 26 | 1 | -2.33 ↓ | -2.33 ↓ | -2.33 ↓ | -1.18 | -1.84 | 2.33 ↑ |
|  |  | 2 | -2.33 ↓ | -2.33 ↓ | -2.33 ↓ | -0.88 | -1.03 | 1.66 |
|  |  | 3 | -2.33 ↓ | -2.05 ↓ | -2.33 ↓ | -1.69 | -0.77 | 2.00 ↑ |
|  | 27 | 1 | -1.64 | -0.92 | -1.88 | 0.05 | 0.70 | 0.00 |
|  |  | 2 | -1.34 | -1.64 | -1.34 | 0.05 | 0.92 | 0.00 |
|  |  | 3 | -2.33 ↓ | -2.05 ↓ | -1.48 | 0.05 | 0.70 | 0.00 |
|  | 28 | 1 | -2.33 ↓ | -1.08 | 1.17 | 1.88 | 1.29 | 1.33 |
|  |  | 2 | -1.75 | -1.08 | 1.23 | 2.03 ↑ | 0.33 | 2.33 ↑ |
|  |  | 3 | -2.33 ↓ | -1.48 | -0.03 | -1.91 | -3.00 ↓ | 1.66 |
|  | 29 | 1 | -2.33 ↓ | -1.88 | -2.33 ↓ | -1.39 | -1.30 | 0.00 |
|  |  | 2 | -2.33 ↓ | -2.33 ↓ | -2.33 ↓ | -1.80 | -0.76 | 1.00 |
|  |  | 3 | -2.33 ↓ | -2.33 ↓ | -2.33 ↓ | -2.51 ↓ | -1.03 | -1.00 |
|  | 30 | 1 | -1.64 | -2.33 ↓ | -1.88 | -2.20 ↓ | -2.35 ↓ | 0.66 |
|  |  | 2 | -2.33 ↓ | -2.33 ↓ | -2.05 ↓ | -2.44 ↓ | -2.35 ↓ | 0.66 |
|  |  | 3 | -2.33 ↓ | -1.88 | -2.33 ↓ | -2.NE80 ↓ | -2.35 ↓ | 0.66 |

RTI, Reaction Time; RVPA, Rapid Visual Information Processing; PAL, Paired Associates Learning; RAVLT-L, Rey's Auditory Verbal Learning Test – Learning (the sum of correctly recalled words across the first five consecutive trials); RAVLT-D, Rey's Auditory Verbal Learning Test – Delayed recall.

^a^The z-scores for the main cognitive outcomes for all participants were calculated using each participant's score, the normative group's mean, and standard deviation. All scores equal to or below -2 are interpreted as below the norm (marked with an arrow down ↓), and equal to 2 and above as above the norm (marked with an arrow up ↑), and all other scores as within the norms.
