# Supplementary material for: Two-year longitudinal neuropsychological monitoring after unilateral and staged bilateral subthalamic nucleus deep brain stimulation
Source: Front Neurosci. 2026 May 8;20:1767180. doi: 10.3389/fnins.2026.1767180 (PMC13194385; doi:10.3389/fnins.2026.1767180)
Supplement: Supplementary file 5 [file Table_5.DOCX]

Table 5. The number of participants below, within, or above the normative samples for the main cognitive outcomes in three assessments.

| **Group** | **Cognitive function** | **Test** | **Below norms**  n (percentage) | | | **Within norms**  n (percentage) | | | **Above norms**  n (percentage) | | |
| --- | --- | --- | --- | --- | --- | --- | --- | --- | --- | --- | --- |
|  |  |  | Pre-DBS  assessment | 6-month  follow-up | 24-month  follow-up | Pre-DBS  assessment | 6-month  follow-up | 24-month  follow-up | Pre-DBS  assessment | 6-month  follow-up | 24-month  follow-up |
| **uDBS**  **n=14** | Psychomotor | RTI | 4 (29%) | 4 (29%) | 5 (36%) | 10 (71%) | 10 (71%) | 9 (64%) | 0 (0%) | 0 (0%) | 0 (0%) |
|  | Attention | RVP | 1 (7%) | 1 (7%) | 1 (7%) | 13 (93%) | 13 (93%) | 13 (93%) | 0 (0%) | 0 (0%) | 0 (0%) |
|  | Memory | PAL | 5 (36%) | 2 (14%) | 3 (21%) | 9 (64%) | 12 (86%) | 11 (79%) | 0 (0%) | 0 (0%) | 0 (0%) |
|  |  | RAVLT-L | 3 (21%) | 4 (29%) | 2 (14%) | 11 (79%) | 10 (71%) | 12 (86%) | 0 (0%) | 0 (0%) | 0 (0%) |
|  |  | RAVLT-D | 2 (14%) | 1 (7%) | 1 (7%) | 12 (86%) | 13 (93%) | 13 (93%) | 0 (0%) | 0 (0%) | 0 (0%) |
|  | Executive functions | Digit Span | 1 (7%) | 0 (0%) | 0 (0%) | 13 (93%) | 13 (93%) | 13 (93%) | 0 (0%) | 1 (7%) | 1 (7%) |
| **bDBS**  **n=16** | Psychomotor | RTI | 9 (56%) | 8 (50%) | 11 (69%) | 7 (44%) | 8 (50%) | 5 (31%) | 0 (0%) | 0 (0%) | 0 (0%) |
|  | Attention | RVP | 2 (12%) | 4 (25%) | 4 (25%) | 14 (88%) | 12 (75%) | 12 (75%) | 0 (0%) | 0 (0%) | 0 (0%) |
|  | Memory | PAL | 4 (25%) | 7 (44%) | 4 (25%) | 12 (75%) | 9 (56%) | 12 (75%) | 0 (0%) | 0 (0%) | 0 (0%) |
|  |  | RAVLT-L | 1 (6 %) | 4 (25%) | 4 (25%) | 15 (94%) | 11 (69%) | 12 (75%) | 0 (0%) | 1 (6 %) | 0 (0%) |
|  |  | RAVLT-D | 3 (19%) | 2 (12%) | 3 (19%) | 13 (81%) | 14 (88%) | 12 (75%) | 0 (0%) | 0 (0%) | 1 (6 %) |
|  | Executive functions | Digit Span | 0 (0%) | 0 (0%) | 0 (0%) | 13 (81%) | 14 (88%) | 13 (81%) | 3 (19%) | 2 (12 %) | 3 (19%) |

uDBS, unilateral Deep Brain Stimulation; bDBS, bilateral Deep Brain Stimulation; RTI, Reaction Time; RVPA, Rapid Visual Information Processing; PAL, Paired Associates Learning; RAVLT-L, Rey's Auditory Verbal Learning Test – Learning (the sum of correctly recalled words across the first five consecutive trials); RAVLT-D, Rey's Auditory Verbal Learning Test – Delayed recall.
